# Critically ill patients with faecal peritonitis: a 5-year review in a tertiary centre

**DOI:** 10.1186/cc14454

**Published:** 2015-03-16

**Authors:** V Paul, A Tridente, P Kaur, M Mahmood, R Mellors, AH Raithatha

**Affiliations:** 1 Sheffield Teaching Hospitals, Sheffield, UK; 2 Whiston Hospital, St Helens & Knowsley, UK

## Introduction

Faecal peritonitis (FP) is a common cause of sepsis and admission to the ICU [1]. We report a review of all patients admitted to our ICU over 5 years with FP. The aim was to define the clinical characteristics, outcomes and risk factors for mortality in ICU patients with FP.

## Methods

Data were extracted retrospectively from electronic case files. The primary outcome was ICU mortality. Secondary outcomes were hospital, 28-day, 90-day and 1-year mortality. Logistic regression analysis was used to identify independent risk factors for mortality.

## Results

Ninety-nine FP patients were admitted between April 2008 and January 2014. Median age was 73 (IQR 61 to 79), with a female preponderance (53.5%). The median ICU length of stay (LOS) was 5 days (IQR 2 to 16). On admission to critical care, clinical data included (all medians): temperature 36.6°C (IQR 36 to 37.2), systolic blood pressure (BP) 113 mmHg (IQR 104 to 136), diastolic BP 56 mmHg (IQR 49 to 67), lactate 2.3 mmol/l (IQR 1.5 to 3.7), bilirubin 12 μmol/l (IQR 9 to 20), haemoglobin 104 g/l (IQR 93 to 116), haematocrit 31 (IQR 28 to 36), creatinine 88 μmol/l (IQR 66 to 152), prothrombin time 13.1 seconds (IQR 11.9 to 14.4). In 86 patients the initial operation was an emergency laparotomy, with primary perforation in 53 cases. Subsequent anastomotic dehiscence and need for relaparotomy happened in 24 and 33 cases respectively. Forty per cent of patients underwent more than one surgical abdominal intervention. The most common antibiotic used was tazobactam and fluconazole was the commonest antifungal. The percentages of patients receiving mechanical ventilation, renal replacement therapy and inotropic/vasopressor support during ICU stay were 72.7%, 25.3% and 84.8% respectively. The ICU and hospital mortality rates were 23.5% and 26.1%, respectively, increasing to 26.7% at 28 days, 28.4% at 90 days and 32.2% at 1 year. None of the surgical factors or diabetes influenced survival. The strongest independent risk factors associated for ICU mortality were systolic BP on ICU admission (OR = 1.05, 95% CI = 1.01 to 1.09, *P *= 0.015), acute kidney injury (AKI) within the first 24 hours of ICU admission (OR = 0.15, 95% CI = 0.03 to 0.9, *P *= 0.026) and lactate on ICU admission (OR = 0.62, 95% CI = 0.39 to 1, *P *= 0.05).

## Conclusion

In this cohort of critically ill FP patients the ICU and 12-month mortality rates were 23.5% and 32.2%, respectively. The most consistent predictors of mortality across all time points were AKI within 24 hours of ICU admission and admission lactate.

## Acknowledgement

VP and AT are joint first authors.
